# The impact of death and caring for the dying and their families on surgeons - an AI assisted systematic scoping review

**DOI:** 10.1186/s12893-025-02792-1

**Published:** 2025-02-05

**Authors:** Jun Rong Tan, Yun Ting Ong, Victoria Jia En Fam, Annushkha Sinnathamby, Nila Ravindran, Yaoyi Ng, Lalit Kumar Radha Krishna

**Affiliations:** 1 Yong Loo Lin School of Medicine, National University of Singapore, NUHS Tower Block, Level 11, Block 1E, Kent Ridge Road, Singapore, 119228 Singapore; 2 Division of Cancer Education, National Cancer Centre Singapore, 30 Hospital Boulevard, Singapore, 168583 Singapore; 3 Department of Psychosocial Oncology, National Cancer Centre Singapore, 30 Hospital Boulevard, Singapore, 168583 Singapore; 4 Khoo Teck Puat National University Children’s Medical Institute, National University Health System, 5 Lower Kent Ridge Road, Singapore, 119074 Singapore; 5 Division of Supportive and Palliative Care, National University Cancer Institute Singapore, 5 Lower Kent Ridge Road, Singapore, 119074 Singapore; 6 Division of Palliative and Supportive Care, National Cancer Centre Singapore, 30 Hospital Boulevard, Singapore, 168583 Singapore; 7 Duke-NUS Medical School, National University of Singapore, 8 College Road, Singapore, 169857 Singapore; 8 Health Data Science, University of Liverpool, Whelan Building, The Quadrangle, Brownlow Hill, Liverpool, Liverpool, L69 3GB UK; 9 Centre for Biomedical Ethics, National University of Singapore, Block MD11, 10 Medical Drive, Singapore, #02-03, 117597 Singapore; 10 Palliative Care Institute Liverpool, Academic Palliative & End of Life Care Centre, Cancer Research Centre, University of Liverpool, 200 London Road, Liverpool, Liverpool, L3 9TA UK; 11 The Palliative Care Centre for Excellence in Research and Education, PalC, Dover Park Hospice, 10 Jalan Tan Tock Seng, Singapore, 308436 Singapore

## Abstract

**Context:**

Surgeons are taking central roles in caring for patients leaving them prone to the emotional turmoil and grief of patients and families and the moral, psychological and existential distress of members of the interprofessional team and trainees. This has implications on patient safety and surgeon welfare.

**Objectives:**

A systematic scoping review was carried out to address the primary research question “what is known of the effects of caring for the dying and the impact of patient’s death on surgeons?”. It is hoped that the insights gained will better guide support and assessment of surgeons in their evolving roles.

**Methods:**

Guided by the Systematic Evidence-based Approach (SEBA), we conducted a systematic scoping review (SSR). This review included articles published between 1st January 2000 and 2nd September 2024 on Pubmed, Embase, Scopus, Google Scholar, ERIC databases. To enhance trustworthiness and enhance the comprehensiveness of our review the articles identified were also evaluated using ChatGPT 4o and Notebook LM. The findings of these assistive processes were compared with the independent thematic and content analysis carried out by the two research teams.

**Results:**

In total, 4966 titles and abstracts were identified, 174 full-text articles were reviewed, and 26 full-text articles analysed. With the findings of the assistive analysis by the AI tools echoing the findings of the research teams- two key domains were identified: (1) the impact on personhood, (2) predisposing factors.

**Conclusion:**

This AI assisted SSR in SEBA confirms that surgeons do suffer from the cumulative effects of caring for dying patients and their families and from the death of the patient and supporting the family and members of the interprofessional team and trainees. Without timely and personalized support surgeons are prone to depression, burnout, and substance abuse, and compromises to patient and family experiences, outcomes, safety and satisfaction. The need for effective longitudinal and personalized assessment tools is clear.

**Supplementary Information:**

The online version contains supplementary material available at 10.1186/s12893-025-02792-1.

## Background

Caring for dying patients and their families precipitates emotional, psychological and existential distress amongst physicians, nurses and medical students [1–9]. In particular, surgeons are taking increasingly central roles in caring for patients and their families and in supporting members of the multidisciplinary team and trainees. It is due to this that the cumulative effects of these emotional, psychological and existential effects of such exposure have come under the spotlight. Recent studies have found caring for terminally ill patients and exposure to death, grief and bereavement impacts how healthcare professionals think, feel and act as professional care providers or their professional identity formation (PIF) [1, 7, 10, 11]. As illustrated by the quote: *“every surgeon carries within himself a small cemetery*,* where from time to time he goes to pray—a place of bitterness and regret*,* where he must look for an explanation for his failures”* [12], supporting surgeons in their expanding roles is key [13, 14].

With implications on patient safety, care provision, patient outcomes, family experiences and surgeon wellbeing [5–7], a systemic scoping review (SSR) of surgeon experiences caring for the terminally ill and their families and dealing with their demise is proposed. It is hoped that the insights will enhance assessment and support of surgeons. To guide address our primary research question - “what is known of the effects of caring for the dying and the impact of patient’s death on surgeons?’ we employ the lens of the Ring Theory of Personhood (RToP) [15].

## The ring theory of personhood (RToP)

**Fig. 1:**
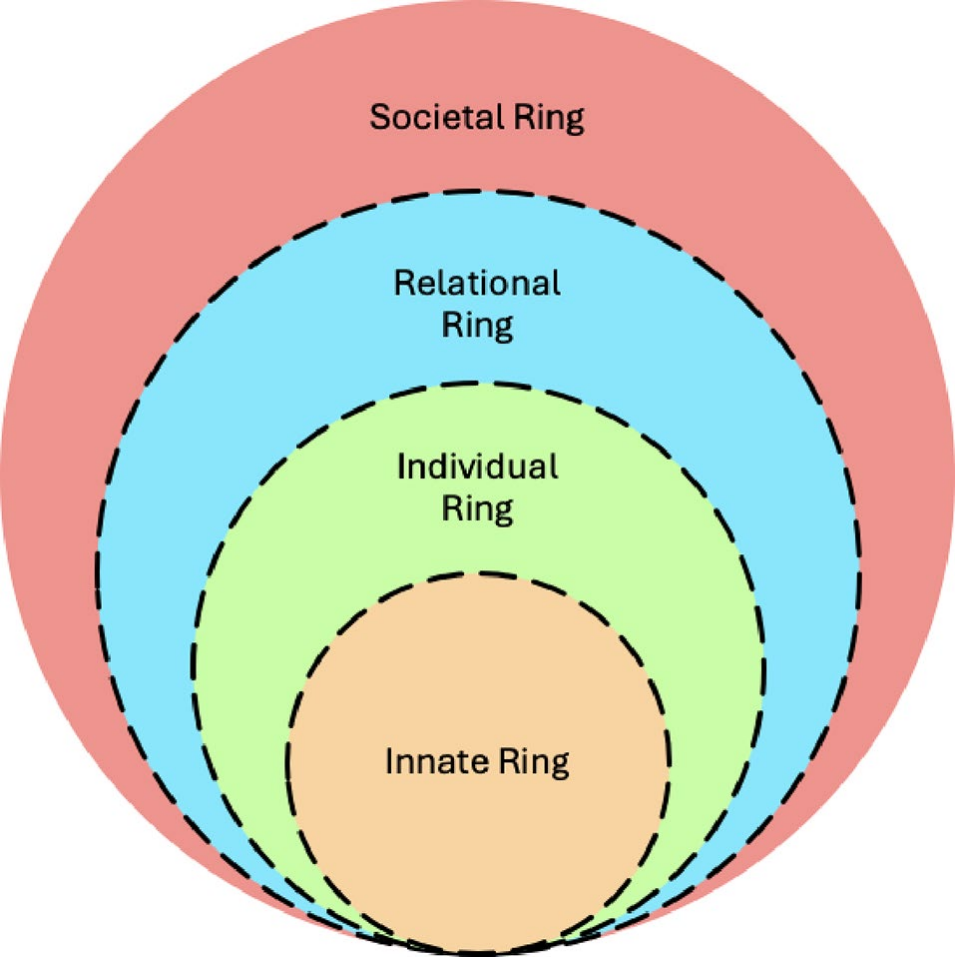
Ring theory of personhood

The employment of the RToP is rooted in the notion that clinical experiences [16, 17], growing competencies, deeper insights, enhanced self-awareness, personalized feedback and guided reflections [18–20] (henceforth nous) impact a surgeon’s beliefs [21], principles, norms, mores, and values (henceforth belief systems) [22]. These changes in belief systems impact a surgeon’s self-concepts of identity and personhood (or what makes you, you). The RToP provides an effective sketch of evolving self-perceptions of personhood and identity where professional identity is an intimately entwined aspect [15, 19, 23–27] Fig. 1.

The RToP posits that belief systems are a combination of Innate, Individual, Relational and Societal beliefs (Table 1) which arise from the Innate, Individual, Relational and Societal Rings which the RToP suggests make up the four key elements of personhood.

Focused upon the physician’s upbringing, demographic features and spiritual and religious beliefs, the Innate Ring forms the backbone of what it means to be human. The Individual Ring is centred on the physician’s conscious function, including their behaviour, emotions and personality. Belief systems guiding close personal relationships with family and friend are housed within the Relational Ring whilst sociocultural, professional, legal and ethical norms, rights, roles and responsibilities form the core of the Societal Ring. The belief systems that stem from these rings are summarised in Table 1.

**Table 1:** The belief systems in each ring of the RToP

Ring of personhood	Description
Innate	Houses belief systems that revolve the narratives created from the physician’s demographic features, and spiritual and religious beliefs.
Individual	Houses belief systems that focus on conscious function and personality.
Relational	Houses belief systems related to close personal relationships, such as family and friends.
Societal	Houses belief systems that pivot on sociocultural, professional, legal and ethical norms, expectations, rights, roles and responsibilities.

Shifts in belief systems, personhood and self-identity brought on by evolving practice, financial, sociocultural and medicolegal and professional expectations are modulated by a surgeon’s nous [23, 24].

Appreciation of this fact in the face of developing nous and changing contextual considerations [28] facilitates timely support and attenuates the development of maladaptive beliefs that negatively impact coping, practice, communication, interprofessional working and patient safety.

### Methods

#### Theoretical lens

We utilised the Systematic Evidence-Based Approach (SEBA) to guide our SSR as its relativist epistemological perspective is especially suited to investigating changes in practice, thinking, perception, belief systems, identity and personhood [29, 30] in the face of caring for the dying and coping with the death of a patient across various surgical settings.

### The systematic evidenced-based approach (SEBA)

**Fig. 2:**
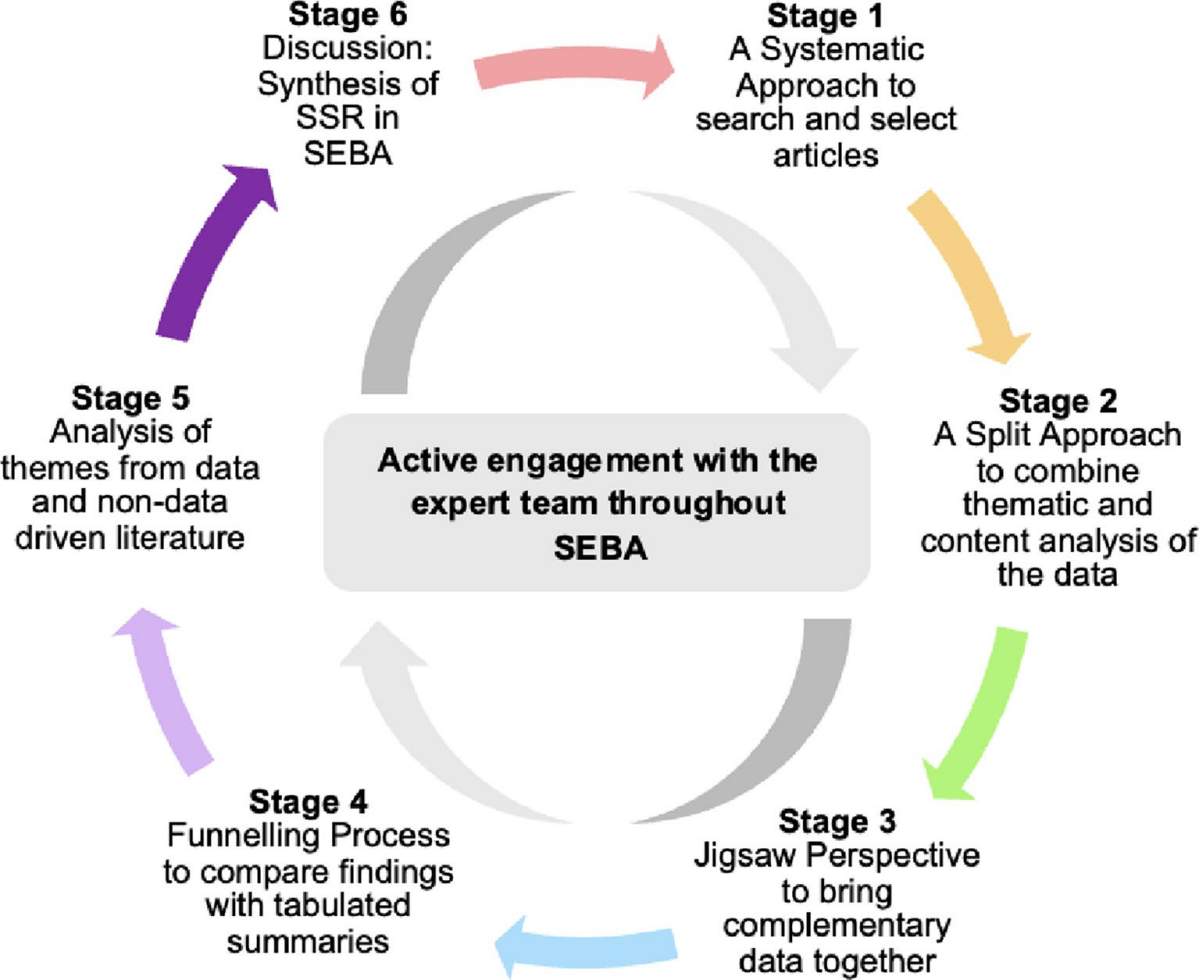
Stages of the systematic evidence-based approach [18, 20, 31]

Complying with the PRISMA-ScR guidelines (see Additional File 1), the six-staged SEBA methodology has continued involvement and consultation of an expert team of medical librarians, local educational experts and clinicians including senior surgeons (Fig. 2) to improve accountability and reproducibility. The SEBA methodology was also reflexive and reiterative.

### Stage 1 of SEBA: The systematic approach

#### i. determining the research question and inclusion criteria

The primary research question was determined to be “what is known of the effects of caring for the dying and the impact of patient’s death on surgeons?”. The research question was guided by a Population, Comparison and Context (PCC) framework (Table 2). Secondary research questions were “how does caring for the dying and contending with the death of a patient impact the personhood of a surgeon?”

**Table 2:** Population, comparison and context (PCC), inclusion Criteria and Exclusion Criteria Applied to Database Search

	Inclusion	Exclusion
Population	Surgeons	Physicians who are not surgeons Non-physicians Medical students
Concept	Impact of exposure to death and dying on the surgeons	NA
Context	All practice settings Articles published within 1 Jan 2000 and 2 Sep 2024	Articles that fall outside of date range Non-English articles

#### i. Searching

Searches for articles published between 1 Jan 2000 and 2 Sep 2024 were conducted on PubMed, Embase, Scopus, Google Scholar, ERIC databases [32]. ‘Snowballing’ through review of the references of included articles identified additional articles [33]. The full search strategy is enclosed in Additional File 2.

#### ii. Extracting and charting

The titles and abstracts were subsequently independently reviewed before discussing their findings for the deconflicting process through Sandelowski and Barroso [34]’s *‘negotiated consensual validation’* that saw *“research team members articulate*,* defend*,* and persuade others of the ‘cogency’ or ‘incisiveness’ of their points of view*” to finalize the list of articles to be included [34]. To minimise bias, equal weight was accorded to each of the research team member during the discussion process to reconcile conflicts. If conflicts were not able to be resolved, the research team then consulted the expert team who provided external third-party guidance. Subsequently, the full text of each article was reviewed to identify the final list of included articles which then underwent quality appraisal (see Additional File 3) [35, 36]. Research team members underwent the same training process for analysis by the senior author to ensure consistent quality appraisal.

### Stage 2 of SEBA: Split approach

The data from the searches were independently and concurrently analysed by two independent teams using the Split Approach with one sub-team utilising Braun and Clarke [37]’s approach to thematic analysis with synthesis of codes from the ‘surface’ meaning of the included articles to arrive at semantic themes [38] and the other employed Hsieh and Shannon [39]’s approach to directed content analysis utilising predetermined codes drawn from Kuek et al. [10]’s ‘*The impact of caring for dying patients in intensive care units on a physician’s personhood: a systematic scoping review’*. Consensus on the key themes and categories of each article was attained through *“negotiated consensual validation”* [34].

### Stage 3 of SEBA: The jigsaw perspective

Resting on the notion that complementary qualitative data gives *“a richer*,* more nuanced understanding of a given phenomenon”* [40] when reviewed together, overlaps in themes and categories were noted as part of the Jigsaw Perspective [41, 42] to form broader themes/categories.

### Stage 4 of SEBA: The funnelling process

In the Funnelling Process, themes/categories were compared with the tabulated summaries of the included articles to ensure that the ‘jigsaw pieces’ appropriately represented the data to form overarching domains.

### Stage 5 of SEBA: analysis of evidence-based and non-data driven literature

Non-peer-reviewed or non-evidence-based literature comprise only a minority of the data sources. By comparing the themes from such literature with that of research-based peer reviewed data, it was ascertained that the research data was not biased through the inclusion of non-peer-reviewed or non-evidence-based literature.

### AI assistance

To enhance reproducibility, trustworthiness and accountability the expert team and research teams drew on ChatGPT and Notebook LM to identify any additional articles, summarize and evaluate the contents of the included articles [43, 44]. The output from ChatGPT and Notebook LM are enclosed in Additional File 4.

## Results

**Fig. 3:**
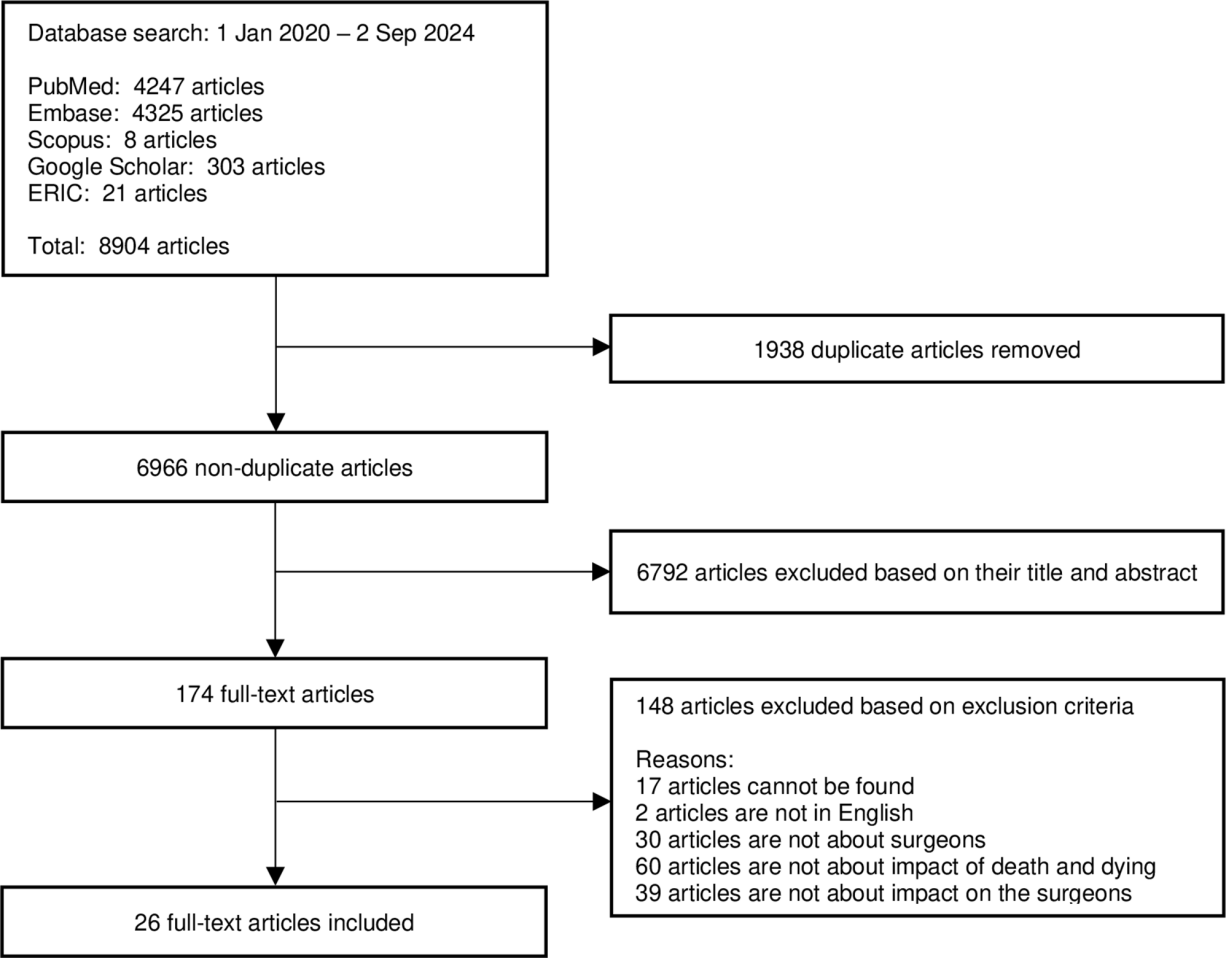
Prisma flowchart

In total, 6966 titles and abstracts were identified after duplicate removal, 174 full-text articles were reviewed, and 26 full-text articles were analysed (Fig. 3.)

The themes identified by Notebook LM were (1) tfhe impact on personhood, (2) implications. The themes identified through ChatGPT were (1) current theories impacting death and dying (2) overarching theories impacting death and dying.

The research and expert teams reviewed the data and AI models’ description of the themes and found that they were in concordance with the main ideas elucidated by the research team. Two key domains were identified: (1) the impact on personhood, (2) predisposing factors.

### Domain 1: impact on personhood

The effects of caring for the dying may be categorised according to the Innate, Individual, Relational and Societal Rings of the RToP.

### i. Innate ring

7 included articles discussed the impact on the Innate Ring. Witnessing a patient’s death sees some surgeons confront their fear of death and sense of helplessness [45, 46]. Precipitating self-reflection and a review of regnant spiritual beliefs and attitudes is often the circumstance of the death [28, 45–48], whether the death was expected [49, 50], dignified, and in a manner that would be consistent with the patient’s wishes [28, 46, 48], was the family adequately prepared and supported and the nature of the relationship [48] shared between surgeon, patient and family.

### Individual ring

18 articles discussed the impact on the Individual Ring. Impact on the individual ring is influenced by background personality traits, self-awareness [14, 50–52], psychological and emotional overlay [14, 46, 53]. This was often tempered by available support [45, 51] and accounts that the ‘labor’ of death [14] was increasingly “shifted out of the operating theatre, off the surgeons’ hands, and onto intensive care physicians, palliative care professionals and nurses”. In truth however Arnold-Foster found that “for many surgeons, emotional detachment is partial or incomplete” and the much vaunted ‘psychological armour’ [51] and detachment [52] expected of surgeons more fragile [14, 53, 54] and regularly pierced making it impossible for a surgeon to disregard the “emotional labour” of death [13, 14, 28, 54, 55].

The effects on the Individual Ring can be immediate, or delayed [53]. Unexpected, and visibly disturbing deaths often leave enduring impressions [14, 46, 53], guilt, and or a sense of professional failure [13, 56]. Acute stress reactions can also arise including sleep disturbances, increased susceptibility to burnout [28] and heightened levels of anxiety and depression and even Post-Traumatic Stress Disorder [13, 14, 28, 45, 52, 54–56].

Delayed effects often follow reflections [13, 57] and impact personal well-being, professional resilience confidence, self-esteem and ego [1, 3, 4, 14]. This may be exacerbated by witnessing the family’s grief [56], medical lawsuits, pressure to succeed in research, financial worries, negative attitudes to gender, and resilience [54] and precipitate burnout [14, 55, 58, 59].

Cumulative effects of patient loss are also described [55]. Hinshaw [45] and Joliat et al. [14] noted that a surgeon’s ego is especially vulnerable in Surgical Palliative Care [14, 46]. Additionally, repeated exposure to loss may drive some individuals toward unhealthy coping mechanisms, such as substance misuse. In extreme cases, these factors can lead to thoughts of self-harm or suicide [13, 14, 28, 45, 52, 54, 56].

### ii. Relational ring

4 articles reported the impact on the Relational Ring. Surgeons report relational [54] and marital difficulties and even divorce as a result of their experiences with death of a patient [49, 52]. Reflecting the wider effects of their experiences, witnessing the suffering of pediatric oncology patients led surgeon’s to become more focused on the well-being of their own children [13].

### iii. Societal ring

17 articles reported the impact on the Societal Ring. Encountering death and dying early in one’s career serve as critical learning opportunities, and role model coping. However poorly managed these experiences [54, 60–64] can lead to a loss of interest, detachment, and compromises to professional standards and standards of care, compromises in patient safety [52, 56], surgeon-patient-family relationships and the therapeutic ties that hinge upon it [13, 28, 48, 56, 57, 65–68]. This is further influenced by the paradigm of “sorting out problems, fixing them, moving on” [13]. Much is shaped by the hidden curricula [45], and little is by way of formal education [54] and psychological support [13, 59].

### Domain 2: predisposing factors

There are several factors predisposing to poor experiences with death and dying.

### i. Culture

10 articles highlighted the crucial role of surgical culture on the impact of death and dying on surgeons. Surgeons are expected to set aside personal responses, maintain composure, show emotional restraint even amid devastating situations [13, 14]. This culture has however been translated in some practices to seeing discussion about feelings as a sign of weakness [13, 45, 56]. The sense of regret and failure [54, 61, 66]. This is compounded in settings where surgery is perceived to be lifesaving and quality enhancing [53], where death may be regarded as a surgical failure [13, 14, 68]. This is also the case in unexpected or sudden deaths [13, 14, 50].

### ii. Practice

13 articles highlighted the impact of surgical practice settings on the impact of death and dying. Poor palliative care training and preparation for patient death is a leading factor in increasing surgeons’ vulnerability to patient mortality [14, 28, 48, 51, 55–57]. Concurrently the notion that the ‘labor of death’ or caring for the dying being managed by other clinical teams also leaves surgeons less likely and capable of confronting its sequelae and the “emotional labour” of death [13, 14, 28, 54, 55]. Indeed, by virtue of the nature of certain surgical subspecialities of relatively lower risk and higher elective caseloads (e.g. orthopedics) that some surgeons have little exposure to death leaving them less experienced and equipped to confront the death of their patients [13, 47]. On the other hand, surgeons participating in high-risk procedures like aortic aneurysm surgeries [13], treatment of critically ill patients and practice in surgical oncology that face more death and dying do not have effective support [13, 48, 51]. These experiences have cumulative effects [55] compounded by overwhelming workloads, long working schedules and the lack of a conducive environment [13, 62] that reduce self-reflection and perpetuates emotional distancing and poor processing of experiences [13, 45, 52, 63, 69].

### iii. Structure

15 articles highlighted that a lack of support at both interpersonal and institutional levels can leave surgeons more vulnerable [47, 60] to the emotional impact of death and dying [58]. Institutionally, there is often an absence of structured or formalized support systems [54–56], such as emotional [58] or mental health resources [14, 55, 68, 69], especially during times when surgeons are most affected by adverse surgical outcomes [13, 14].

Morbidity and mortality conferences that place excessive emphasis on clinical aspects of patient death are recognized as another contributing factor [50, 51]. Many are adversarial and lack psychological safety hindering effective sharing and debriefs [13, 45, 56, 62].

## Discussion

In answering its primary research question this SSR in SEBA highlights the cumulative effects of patient death and caring for a dying patient, their grieving family, the interprofessional team and trainees (henceforth death and dying) on surgeons. These include short and long term ill-effects such as depression, burnout, and substance abuse [52], and compromises patient and family experiences, outcomes, safety and satisfaction. These findings align with themes observed in their medical counterparts, who also face the emotional toll of death and dying. In particular - chronic exposure to death leads to emotional fatigue and burnout, especially amongst surgeons lacking resilience and displaying poor coping especially when patients’ deaths were traumatic, unexpected and the surgeon lacked holistic and longitudinal support in a culture where vulnerability is seen as weakness [14, 28, 45, 46, 53, 58]. This is especially concerning in surgical practice settings where individual risks and responsibilities may be arguably more significant [13, 48, 51].

**Fig. 4:**
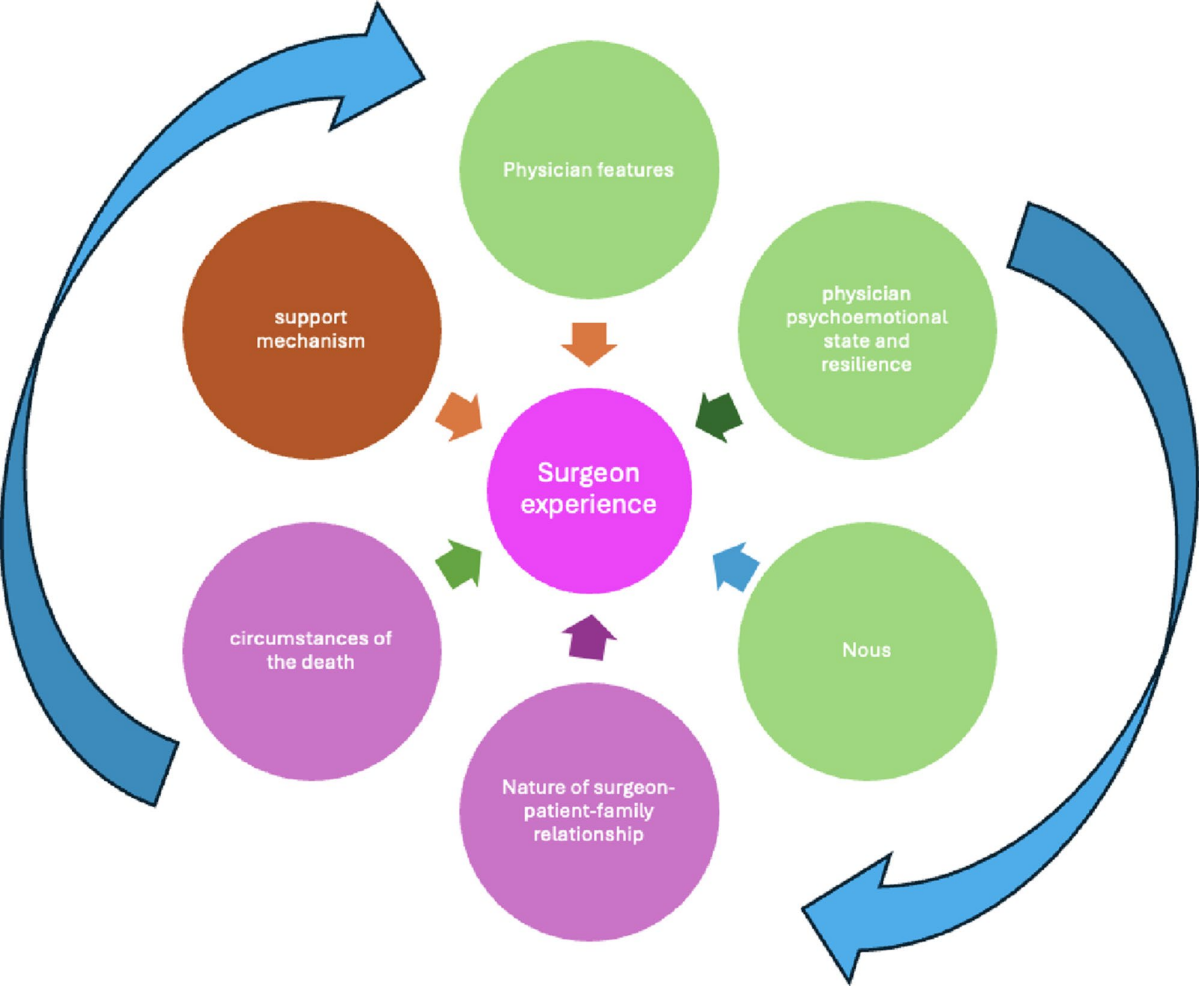
Factors influencing the impact of death and dying on surgeons

This SSR in SEBA shed insights into the impact of death and dying suggesting that their impact on a surgeon may be explained by Fig. 4. This personalized perspective acknowledges the physician’s characteristics, psycho-emotional state, resilience, ability and willingness to reflect on their experiences and the lens through which they make sense of these experiences, emotions, feedback and reflections. It also recognizes that personalized responses are tempered by the surgeon’s nous, motivation and ability to garner support. It is the surgeon’s sense of self or personhood that influences their responses. Further, this model accommodates contextual factors such as the specific circumstances of the patient’s death, the nature, duration, personalization, and setting in which the surgeon, patient and family interact.

These interactions, effects and responses are not static but constantly evolving highlighted by the arrows surrounding Fig. 4. Affect, setting, context [28], developing nous, maturity, shifting resilience, accessibility of support structures and mechanisms, the presence of a conducive environment and the evolving nature of relationships between surgeon, patient, family and team introduce a level of complexity in these considerations not previously discussed. As linear cause and effect-based models give way to more complicated notions, how we consider the effects of death and dying on surgeons must also evolve.

In the face of such complexity focus should be on three considerations– the surgeon and his personhood, support structured and the practice culture. Surgeons should be better prepared for their expanding role in holistic patient centered multi-disciplinary care. Training in palliative and end of life care skills and competencies, team management and interprofessional working to support patients and family have become requisite competencies in surgical training though consistent implementation has yet to be achieved [70–72]. Work is still required to ensure holistic, longitudinal structured training such as through formal, accessible training programmes, workshops and mentorship is incorporated into continuing surgical education. Mentorship in particular and its capacity for personalized, appropriate and longitudinal support that can accommodate variabilities in surgeon and contextual factors may play a key role in supporting junior surgeons [2, 4, 27, 73, 74]. Portfolios may also serve as a useful tool in capturing ongoing evolution of individual and contextual factors [30, 75–77]. Part of this process of supporting surgeons must be in ensuring that practice culture continues to evolve towards a conducive, reflective practice culture requiring commitment from key decision makers such as through shifting morbidity and mortality rounds away from a culture of blame but towards a culture of growth [78].

### Limitations

Limitations of this study included a predominance of American and European literature, which may limit the generalizability of the study on other contexts with their unique sociocultural and practice factors. Only English articles were included which may have resulted in the exclusion of relevant non-English articles. Despite efforts to reduce biases through the negotiated consensual approach and engagement of the expert team, individual bias may still persist. Use of AI in literature reviews is also novel and challenges in ensuring accuracy and reducing machine bias may still remain. The research team has attempted to address this bias by limiting the use of AI as a means to countercheck and augment the team’s work instead of replacing it completely.

## Conclusion

This AI-assisted SSR in SEBA highlights the deep impact of caring for the dying and confronting patient death on surgeons’ sense of self, personhood, and identity. While these findings align with evidence from literature on the impact of death and dying on physicians, further research is needed to fully elucidate its implications for patient care, safety, and the evolving identity of surgeons. This includes ensuring longitudinal assessments to guide support and practice, structuring an effective training environment replete with effective culture and a mentoring program that runs alongside the surgeon throughout their training career.

## Electronic supplementary material

Below is the link to the electronic supplementary material.

Supplementary Material 1

Supplementary Material 2

Supplementary Material 3

Supplementary Material 4

Supplementary Material 5

## Data Availability

The authors confirm that the data supporting the findings of this study are available within the article and its supplementary materials. To request data from this study, please contact Prof Lalit Kumar Radha Krishna (lalit.radha-krishna@liverpool.ac.uk).

## Abbreviations

SEBA: Systematic evidence-based approach
AI: Artificial intelligence
SSR: Systematic scoping review
PIF: Professional identity formation
RtoP: Ring theory of personhood
PCC: Population, comparison, context
MERSQI: Medical education research study quality instrument
COREQ: Consolidated criteria for reporting qualitative studies
