# Supplementary material for: The impact of death and caring for the dying and their families on surgeons - an AI assisted systematic scoping review
Source: BMC Surg. 2025 Feb 5;25:56. doi: 10.1186/s12893-025-02792-1 (PMC11796083; doi:10.1186/s12893-025-02792-1)
Supplement: Supplementary file 1 — Supplementary Material 1 [file 12893_2025_2792_MOESM1_ESM.docx]

**Additional File 2: Full Search Strategy**

**Pubmed**

("Death"[MeSH] OR "Terminal Care"[MeSH] OR "Attitude to Death"[MeSH] OR "Palliative Care"[MeSH] OR death[tiab] OR dying[tiab] OR die[tiab] OR end-of-life[tiab] OR "end of life"[tiab] OR “pass on”[tiab])

AND

("Surgeons"[MeSH] OR "General Surgery"[MeSH] OR surg*[tiab])

AND

("Stress, Psychological"[MeSH] OR "Mental Health"[MeSH] OR "Attitude"[MeSH] OR "Perception"[MeSH] OR "Cognitive Reflection"[MeSH] OR "Mindfulness"[MeSH] OR "Thinking"[MeSH] OR impact*[tiab] OR effect*[tiab] OR affect*[tiab] OR consequen*[tiab] OR influen*[tiab] OR burden*[tiab] OR stress*[tiab] OR toll[tiab] OR "psychological effect*"[tiab] OR "emotional effect*"[tiab] OR "emotional impact"[tiab] OR "mental health"[tiab] OR "psychological distress"[tiab] OR coping*[tiab] OR burnout[tiab] OR attitude*[tiab] OR view*[tiab] OR perception*[tiab] OR perceive*[tiab] OR reflect*[tiab] OR factor*[tiab] OR mindful*[tiab])

AND

("Behavior and Behavior Mechanisms"[MeSH] OR "Personhood"[MeSH] OR "Interpersonal Relations"[MeSH] OR "Spirituality"[MeSH] OR "Religion"[Mesh] OR "Professional Role"[MeSH] OR "Cognition"[MeSH] OR "Fatigue"[MeSH] OR behavior*[tiab] OR personhood[tiab] OR relationship*[tiab] OR spiritual*[tiab] OR relig*[tiab] OR "professional role"[tiab] OR cognition[tiab] OR "cognitive function"[tiab] OR fatigue*[tiab] OR burnout*[tiab] OR burntout[tiab] OR "burnt out"[tiab] OR identit*[tiab] OR "sense of self"[tiab])

NOT

("Nurses"[MeSH] OR "Pharmacists"[MeSH] OR "Pharmacy"[MeSH] OR "Dentists"[MeSH] OR "Dental Health Services"[MeSH] OR "Allied Health Personnel"[MeSH] OR nurs*[tiab] OR pharmac*[tiab] OR dentist*[tiab] OR dental*[tiab] OR "allied health"[tiab])

NOT

("Anesthesiology"[MeSH] OR "Anesthesiologists"[MeSH] OR "Anesthesia"[MeSH] OR "Critical Care"[MeSH] OR "Intensive Care Units"[MeSH] OR anaesthes*[tiab] OR anesthes*[tiab] OR intensivist*[tiab] OR "critical care"[tiab] OR "intensive care"[tiab] OR ICU[tiab])

NOT

("Pediatrics"[MeSH] OR "Pediatricians"[MeSH] OR "Infant, Newborn"[MeSH] OR "Infant"[MeSH] OR "Child, Preschool”[MeSH] OR "Child"[MeSH] OR "Adolescent"[MeSH] OR pediatric*[tiab] OR paediatric*[tiab] OR infant*[tiab] OR child*[tiab] OR adolescen*[tiab] OR youth*[tiab] OR teen*[tiab])

NOT

("Obstetrics"[MeSH] OR "Gynecology"[MeSH] OR "Obstetrics and Gynecology Department, Hospital"[MeSH] OR "Obstetricians"[MeSH] OR "Gynecologists"[MeSH] OR "Pregnancy"[MeSH] OR "Pregnant Women"[MeSH] OR "Prenatal Care"[MeSH] OR "Perinatal Care"[MeSH] OR "Postnatal Care"[MeSH] OR obstetr*[tiab] OR gynecol*[tiab] OR gynaecol*[tiab] OR obstetrician*[tiab] OR gynecologist*[tiab] OR gynaecologist*[tiab] OR pregnan*[tiab] OR childbirth[tiab] OR prenatal[tiab] OR perinatal[tiab] OR postnatal[tiab])

**Embase**

('death'/mj OR 'terminal care'/mj OR 'attitude to death'/mj OR death:ab,ti OR dying:ab,ti OR die:ab,ti OR 'end-of-life':ab,ti OR 'end of life':ab,ti OR 'pass on':ab,ti)

AND

('surgeon'/mj OR 'general surgery'/mj OR surg*:ab,ti)

AND

('physiological stress'/mj OR 'mental health'/mj OR 'attitude'/mj OR 'perception'/mj OR 'cognitive reflection'/mj OR 'mindfulness'/mj OR 'thinking'/mj OR impact*:ab,ti OR effect*:ab,ti OR affect*:ab,ti OR consequen*:ab,ti OR influen*:ab,ti OR burden*:ab,ti OR stress*:ab,ti OR toll:ab,ti OR 'psychological effect*':ab,ti OR 'emotional effect*':ab,ti OR 'emotional impact':ab,ti OR 'mental health':ab,ti OR 'psychological distress':ab,ti OR coping*:ab,ti OR burnout:ab,ti OR attitude*:ab,ti OR view*:ab,ti OR perception*:ab,ti OR perceive*:ab,ti OR reflect*:ab,ti OR factor*:ab,ti OR mindful*:ab,ti)

AND

('behavior'/mj OR 'personhood'/mj OR 'human relation'/mj OR 'religion'/mj OR 'professional standard'/mj OR 'cognition'/mj OR 'fatigue'/mj OR behavior*:ab,ti OR personhood:ab,ti OR relationship*:ab,ti OR spiritual*:ab,ti OR relig*:ab,ti OR 'professional role':ab,ti OR cognition:ab,ti OR 'cognitive function':ab,ti OR fatigue*:ab,ti OR burnout:ab,ti OR burntout:ab,ti OR 'burnt out':ab,ti OR identit*:ab,ti OR 'sense of self':ab,ti)

NOT

('nurse'/exp OR 'nurse attitude'/exp OR 'pharmacist'/exp OR 'pharmacy (shop)'/exp OR 'pharmacist attitude'/exp OR 'dentist'/exp OR 'dental procedure'/mj OR 'paramedical personnel'/mj OR nurs*:ab,ti OR pharmac*:ab,ti OR dentist*:ab,ti OR dental*:ab,ti OR 'allied health':ab,ti)

NOT

('anesthesiology'/exp OR 'anesthesiologist'/exp OR 'anesthesia'/exp OR 'intensive care'/exp OR 'intensive care unit'/exp OR anaesthes*:ab,ti OR anesthes*:ab,ti OR intensivist*:ab,ti OR 'critical care':ab,ti OR 'intensive care':ab,ti OR icu:ab,ti)

NOT

('pediatrics'/exp OR 'pediatrician'/exp OR 'infant'/exp OR 'child'/exp OR 'adolescent'/exp OR pediatric*:ab,ti OR paediatric*:ab,ti OR infant*:ab,ti OR child*:ab,ti OR adolescen*:ab,ti OR youth*:ab,ti OR teen*:ab,ti)

NOT

('obstetrics'/exp OR 'gynecology'/exp OR 'obstetrician'/exp OR 'gynecologist'/exp OR 'pregnancy'/exp OR 'pregnant woman'/exp OR 'prenatal care'/exp OR 'perinatal care'/exp OR 'postnatal care'/exp OR obstetr*:ab,ti OR gynecol*:ab,ti OR gynaecol*:ab,ti OR obstetrician*:ab,ti OR gynecologist*:ab,ti OR gynaecologist*:ab,ti OR pregnan*:ab,ti OR childbirth:ab,ti OR prenatal:ab,ti OR perinatal:ab,ti OR postnatal:ab,ti)

**Scopus**

TITLE-ABS-KEY(

(death OR "terminal care" OR "attitude to death" OR "palliative care" OR dying OR die OR "end-of-life" OR "end of life" OR "pass on")

W/5

("psychological stress" OR "mental health" OR attitude OR perception OR "cognitive reflection" OR mindfulness OR thinking OR impact* OR effect* OR affect* OR consequen* OR influen* OR burden* OR stress* OR toll OR "psychological effect*" OR "emotional effect*" OR "emotional impact" OR "mental health" OR "psychological distress" OR coping* OR burnout OR attitude* OR view* OR perception* OR perceive* OR reflect* OR factor* OR mindful*)

W/5

(behavior OR "behavior mechanisms" OR personhood OR "interpersonal relations" OR spirituality OR religion OR "professional role" OR cognition OR fatigue OR relationship* OR spiritual* OR relig* OR "professional role" OR cognition OR "cognitive function" OR fatigue* OR burnout OR burntout OR "burnt out" OR identit* OR "sense of self")

W/5

(surgeon* OR surgery* OR "general surgery")

AND NOT

(nurse* OR pharmac* OR dentist* OR dental* OR "allied health")

AND NOT

(anaesthes* OR anesthes* OR intensivist* OR "critical care" OR "intensive care" OR ICU)

AND NOT

(pediatric* OR paediatric* OR infant* OR child* OR adolescen* OR youth* OR teen*)

AND NOT

(obstetr* OR gynecol* OR gynaecol* OR obstetrician* OR gynecologist* OR gynaecologist* OR pregnan* OR childbirth OR prenatal OR perinatal OR postnatal))

**Google Scholar**

Intitle:("Death" OR "Terminal Care" OR "Attitude to Death" OR "Palliative Care" OR death OR dying OR "end-of-life" OR "end of life" OR "pass on")

AND

Intitle:(surgeon OR "General Surgery" OR surgery)

AND

("Psychological Stress" OR "Mental Health" OR Attitude OR Perception OR "Cognitive Reflection" OR Mindfulness OR Thinking OR impact OR effect OR affect OR consequence OR influence OR burden OR stress OR toll OR "psychological effect" OR "emotional effect" OR "emotional impact" OR "mental health" OR "psychological distress" OR coping OR burnout OR attitude OR view OR perception OR perceive OR reflect OR factor OR mindful)

AND

(behavior OR "Behavior Mechanisms" OR personhood OR "Interpersonal Relations" OR spirituality OR religion OR "Professional Role" OR cognition OR fatigue OR relationship OR spiritual OR religious OR "professional role" OR cognition OR "cognitive function" OR fatigue OR burnout OR identity OR "sense of self")

-Nurses -Pharmacists -Pharmacy -Dentists -"Dental Health Services" -"Allied Health Personnel" -nurse -pharmac -dentist -dental -"allied health" -Anesthesiology -Anesthesiologists -Anesthesia -"Critical Care" -"Intensive Care Units" -anaesthesia -anesthesia -intensivist -"critical care" -"intensive care" -ICU -Pediatrics -Pediatricians -Infant -Child -Adolescent -pediatric -paediatric -infant -child -adolescent -youth -teen -Obstetrics -Gynecology -obstetrics -gynecology -obstetrician -gynecologist -pregnancy -prenatal -perinatal -postnatal

**ERIC**

(descriptor:"Death" OR descriptor:"Hospices (Terminal Care)" OR (title:death OR abstract:death) OR (title:dying OR abstract:dying) OR (title:die OR abstract:die) OR (title:end-of-life OR abstract:end-of-life) OR (title:"end of life" OR abstract:"end of life") OR (title:"pass on" OR abstract:"pass on"))

AND

(descriptor:"Surgery" OR (title:surg* OR abstract:surg*))
