# Supplementary material for: The impact of death and caring for the dying and their families on surgeons - an AI assisted systematic scoping review
Source: BMC Surg. 2025 Feb 5;25:56. doi: 10.1186/s12893-025-02792-1 (PMC11796083; doi:10.1186/s12893-025-02792-1)
Supplement: Supplementary file 2 — Supplementary Material 2 [file 12893_2025_2792_MOESM2_ESM.docx]

**AI Assistance Software:** Notebook LM

**Input:** Uploaded all the PDF documents of included articles

**Prompt:** Please summarise the following articles

**Output:**
Here are summaries of the articles you provided, focusing on the topic of surgeons and patient death.

●

**'A small cemetery'- death and dying in the contemporary British operating theatre.pdf'**: This article explores the emotional impact of patient death on surgeons, arguing that it's often underestimated. It examines how surgeons use narratives of death to construct their professional identity. The article notes that the "labor" of death has shifted away from surgeons and onto other healthcare professionals, such as those in intensive care and palliative care. The article uses surgeon interviews and surgical memoirs to examine how some deaths are remembered while others are forgotten. It concludes by reflecting on the support systems in place for surgeons to manage grief, loss, and potential trauma.1

●

**'02 jamasurgery_srinivasa_2019_rv_180012.pdf'**: This systematic review examines the consequences of patient complications for surgeons, focusing on their emotional responses, coping strategies, and available support mechanisms. The review included nine studies, analyzing the impact of complications on the surgeons' professional and personal lives. The review identified four key themes: (1) adverse emotional consequences of complications, (2) coping mechanisms used by surgeons and trainees, (3) institutional support mechanisms and barriers to support, and (4) consequences of complications for future clinical practice. The findings suggest a need for enhanced support systems at various levels to address the psychological well-being of surgeons facing patient complications.234

●

**'**[**10.1002@bjs.11452.pdf**](mailto:10.1002@bjs.11452.pdf)**'**: This study investigates the prevalence of regret among surgeons following patient deaths. It analyzed responses from a survey of surgeons regarding what could have been done differently in cases where patients died. The analysis, conducted by social scientists and surgeons, identified two main themes: dilemmas and difficult decisions, and communication matters. The study concludes that regret is a significant issue for surgeons, stemming from challenging clinical decisions and communication complexities.56

●

**'Cancer surgeons' distress and well-being, II- modifiable factors and the potential for organizational interventions.pdf'**: This study builds upon a previous paper that identified a high prevalence of burnout and psychiatric morbidity among surgical oncologists. This paper focuses on examining modifiable workplace factors and other stressors that contribute to burnout, psychiatric morbidity, and low quality of life among these surgeons. The study also explores the surgeons' interest in potential interventions to reduce distress and improve their well-being.7

●

**'Deciphering the surgeons' stories.pdf'**: This commentary analyzes a collection of narratives written by surgeons about ethical decision-making. It identifies key themes, including the preponderance of end-of-life issues, the struggle to maintain empathy in challenging circumstances, and disagreements among colleagues about patient management in situations of medical uncertainty. The commentary also calls for more research to explore missing themes and perspectives in surgical narratives.89

●

**'Is a foundation interim year one post better for improving confidence in the care of the dying in general surgery in a tertiary centre or a district general hospital.pdf'**: This study focuses on the impact of a foundation interim year (FiY1) post on the confidence of junior doctors (FY1s) in caring for dying patients. It compares the confidence levels of FY1s who completed a FiY1 post in a tertiary center versus a district general hospital. The study found that FiY1 posts, while beneficial, could be improved to standardize the experience and enhance confidence in all areas related to end-of-life care.1011

●

**'Spiritual dimensions of surgical palliative care.pdf'**: This source emphasizes the significance of spiritual dimensions within surgical palliative care. It highlights how spirituality, distinct from religiosity, provides meaning and purpose to life. The source underscores the need for sensitivity to spiritual issues that often arise at the end of life and advocates for incorporating spiritual history into patient care.12

●

**'Surgeon Perspectives Regarding Death and Dying.pdf'**: This study examines the perspectives of surgeons regarding death and dying, particularly focusing on barriers to providing palliative and end-of-life care. It uses a mixed-methods approach, including surveys and qualitative analysis, to explore the challenges surgeons face in addressing these sensitive aspects of care.1314

●

**'Surgeon and medical student response to patient death.pdf'**: This study investigates how surgeons and medical students respond to patient death, assessing their experiences, coping mechanisms, and associated symptoms. The study highlights the emotional toll of patient death on healthcare professionals and the need for support systems to address their well-being. It found that while formal memorial services can offer support, more tailored interventions are needed to promote healthy coping strategies and mitigate the risk of burnout and distress.15161718

●

**'Surgery Residents' Experiences With Seriously-Ill and Dying Patients- An Opportunity to Improve Palliative and End-of-Life Care.pdf'**: This qualitative study investigates surgery residents’ experiences with seriously ill and dying patients, focusing on their attitudes toward palliative care, their knowledge of this specialty, and the training they receive within the surgical culture. It highlights the challenges residents face in navigating palliative and end-of-life care within a surgical context.1920

●

**'Systematic review of the impact of patient death on surgeons.pdf'**: This systematic review synthesizes existing literature on how surgeons cope with patient death. The review identified seven relevant studies, highlighting the significant psychological burden associated with patient mortality. The findings emphasize the need for greater awareness of the impact of patient death on surgeons and the development of effective support mechanisms to address their psychological well-being.21222324

●

**'The surgeon and palliative care.pdf'**: This article advocates for integrating palliative care into surgical practice, highlighting the importance of addressing the needs of patients with advanced illnesses. The authors emphasize the need for clear definitions of surgical palliation, enhanced funding for research, and standardized tools for assessing quality of life and the impact of interventions.2526

●

**'The surgeon-patient relationship in advanced illness.pdf'**: This article explores the complexities of the surgeon-patient relationship in the context of advanced illness. It highlights the importance of effective communication and empathetic care when navigating end-of-life discussions and decisions.2728

●

**'Unexpected intraoperative patient death- the imperatives of family- and surgeon-centered care.pdf'**: This article examines the challenges of unexpected intraoperative patient death, focusing on the communication and support needs of both the family and the surgeon. It provides guidelines for breaking bad news and emphasizes the importance of honesty, empathy, and self-care for surgeons facing this difficult situation.29303132

●

**'What we still don't know about surgeon response to patient death.pdf'**: This commentary reflects on a study about surgeon and medical student responses to patient death, highlighting the need for further research in this area. It underscores the prevalence of burnout among surgeons stemming from patient deaths and advocates for interventions like memorial services to promote healthy coping mechanisms and foster camaraderie within surgical departments.3334

●

**'Your best life- dealing with loss.pdf'**: This source, while focusing broadly on well-being for surgeons and scientists, acknowledges the importance of dealing with loss in maintaining a balanced life. It implies that coping with the death of patients is a significant aspect of surgical practice.3536

●

**'angelos2012.pdf'**: This article, using vignettes presented to surgeons, explores the influence of factors like the nature of the surgery (emergency vs. elective) and surgeon error on postoperative decision-making. The article highlights the impact of these factors on the surgeon-patient relationship and the practice of surgery.3738

●

**'apm-12-01-70.pdf'**: This study investigates surgeon and medical student responses to patient death. It describes the use of an annual patient memorial service in the department of surgery as a potential intervention to address burnout and emotional distress among healthcare professionals.3940414243

●

**'chamberlain2019.pdf'**: This article explores the associations between end-of-life discussions, patient mental health, medical care near death, and caregiver bereavement adjustment. While it doesn't focus specifically on surgeons, it touches on the broader context of end-of-life care in which surgeons operate.44

●

**'dillon2018.pdf'**: This study examines surgeon perspectives regarding death and dying, focusing on their perceived barriers to providing palliative and end-of-life care. It uses a mixed-methods approach to explore the factors influencing surgeon involvement in these aspects of patient care.4546

●

**'fsurg-09-898274.pdf'**: This study investigates the impact of patient loss on surgeons’ psychosocial well-being and professional careers. The findings suggest that patient death negatively affects surgeons’ emotional and professional lives, underscoring the need for support systems to help them cope with the stress.4748

●

**'jalili-et-al-2023-investigating-surgeons-reactions-to-patients-death-a-phenomenological-study.pdf'**: This phenomenological study examines surgeons’ reactions to patient death, exploring their emotional, mental, and behavioral responses. The study identifies themes of emotional distress, coping mechanisms, and potential post-traumatic growth following patient death.49505152

●

**'joliat2019.pdf'**: This correspondence responds to a commentary on a systematic review about the impact of patient death on surgeons. It discusses the second victim phenomenon and the potential for defensive medicine as responses to the emotional burden and legal risks associated with patient mortality.53

●

**'oskrochi2016.pdf'**: This systematic review and meta-analysis investigates the prevalence of burnout among surgeons, exploring factors that contribute to this phenomenon. The review identifies a range of prevalence rates across surgical specialties, highlighting the widespread nature of burnout in the profession.54555657...

●

**'suwanabol2020.pdf'**: This qualitative study examines surgery residents' experiences with seriously ill and dying patients. The study explores their understanding of palliative care, their training experiences, and the cultural influences within surgical training that shape their perspectives on end-of-life care.6162

●

**'tarpley2011.pdf'**: This source focuses on the spiritual dimensions of surgical palliative care, emphasizing the importance of addressing the spiritual needs of patients facing end-of-life situations. It provides a framework for incorporating spiritual care into the surgical context.63

●

**'taylor2013.pdf'**: This article proposes an integrated curriculum to enhance surgeons’ communication skills when informing families about unexpected patient deaths. The curriculum aims to develop proficiency, competence, confidence, and composure in delivering such sensitive information.6465666768

Surgeons & Patient Death: Examining Personhood Transformation Through The Ring Theory of Personhood (RToP)

The sources offer a rich exploration of how surgeons experience and process patient death, highlighting changes in their beliefs and identity that align with the RToP.

The Innate Ring: Shifting Foundations

●

**Early Exposure to Death and Dying:** The sources describe how surgeons are often exposed to death early in their careers, shaping their understanding of mortality12. These experiences can profoundly impact their Innate Ring, challenging pre-existing beliefs about life, death, and the role of medicine.

●

**Spiritual and Religious Beliefs as a Foundation:** While not explicitly discussed in detail, the sources acknowledge the potential influence of spiritual and religious beliefs on surgeons' perspectives on death34. These belief systems, often rooted in the Innate Ring, can either provide solace or create conflict in navigating these experiences.

The Individual Ring: Emotional and Behavioral Shifts

●

**Emotional Toll and Coping Mechanisms:** The sources strongly emphasize the emotional toll patient death takes on surgeons, even if it's not as profound as the family's grief156. They describe various coping mechanisms employed, including:

○

**Detachment:** Attempting to distance themselves from the emotional intensity of the situation to maintain professional composure78.

○

**Rationalization:** Reframing death as a natural part of the surgical process or focusing on the technical aspects of the case9.

○

**Seeking Meaning:** Finding a sense of purpose in their work and the patient's life to mitigate feelings of loss10.

●

**The "Surgical Personality" in Question:** The sources also question the traditional "surgical personality" characterized by action-orientation and stoicism111213. They suggest that this persona can hinder introspection and emotional processing, ultimately affecting surgeons' well-being and their ability to connect with patients and families.

The Relational Ring: Impact on Personal Relationships

●

**Work-Life Conflict and Strain on Relationships:** While not a primary focus, the sources acknowledge the potential strain of a surgical career on personal relationships. Burnout, long work hours, and emotional exhaustion can negatively impact family life and friendships14. This suggests a potential tension between the demands of the Societal Ring (professional responsibilities) and the Relational Ring (personal connections).

●

**Support from Loved Ones as a Buffer:** The sources highlight the importance of spousal and familial support in mitigating the negative effects of the profession14. Strong relationships within the Relational Ring can provide a crucial buffer against the emotional challenges of dealing with death and the demands of a surgical career.

The Societal Ring: Navigating Professional Norms and Expectations

●

**Shifting Paradigms in Surgical Culture:** The sources point to a potential shift in the Societal Ring surrounding surgical practice151617. They discuss the growing recognition of:

○

**The Importance of Palliative Care:** The need for surgeons to be equipped to address end-of-life care and support patients beyond the operating room618.

○

**Open Communication and Empathy:** The value of open communication with patients, families, and colleagues, and the importance of empathy in the surgeon-patient relationship51920.

●

**Need for Training and Support Systems:** The sources advocate for changes in surgical training and the development of support systems to address the emotional needs of surgeons212223. This reflects a call for a more holistic approach to professional development within the Societal Ring, one that recognizes the human impact of the work.

Further Insights Through the RToP

●

**Transformation of Personhood:** The RToP helps us understand how experiences with death and dying can profoundly alter a surgeon's concept of personhood. It's not just about emotional responses; it's about a fundamental shift in their beliefs, values, and how they define themselves as surgeons and individuals.

●

**Interconnectedness of the Rings:** The sources, analyzed through the RToP, reveal the complex interplay between the different rings. A surgeon's Innate beliefs shape their Individual responses, which then affect their Relational and Societal interactions. Each ring is intrinsically linked and impacts the others.

Gaps and Areas for Further Research

●

**Longitudinal Studies:** While the sources highlight the immediate and short-term impact of patient death, more research is needed on how these experiences shape surgeons over their entire careers.

●

**Impact of Specific Death Circumstances:** The sources don't extensively explore how different circumstances surrounding death (e.g., unexpected intraoperative death vs. death after a prolonged illness) might differentially affect the rings of personhood.

●

**Cultural Influences:** The sources primarily focus on Western surgical contexts. More research is needed on how different cultural beliefs and practices might influence surgeons' experiences and coping mechanisms.

By applying the RToP, the sources offer a nuanced understanding of how patient death impacts surgeons beyond immediate emotional responses. It reveals the profound and often transformative changes in their sense of self, affecting all aspects of their being—from their core beliefs to their relationships and professional identity. This framework can guide future research and inform the development of support systems to address the complex needs of surgeons navigating the profound human experiences inherent in their profession.

**AI Assistance Software:** ChatGPT

**Input:** Uploaded all PDFs of included articles in the SSR

**Prompt:** Please summarise the following articles

**Output:**

Death and dying papers summary and theories

1. **Joliat, Demartines, & Uldry (2019)**
   - **Summary**: This systematic review examines how patient death impacts surgeons, highlighting the emotional and psychological toll. Surgeons often experience psychological distress, including burnout, as patient death is perceived as a professional failure due to their goal-oriented training (Joliat et al., 2019)​(10.1002@bjs.11264).
   - **Theories Used**: Psychological distress and burnout theories, coping mechanisms in high-stress professions.
2. **Page (2011)**
   - **Summary**: This article delves into the need for introspection among surgeons, who often resist self-reflection due to the “surgical personality” that emphasizes detachment and resilience. Page suggests that introspection may help surgeons manage the emotional burden of patient death (Page, 2011)​(08 page).
   - **Theories Used**: Introspection and self-reflection theories; professional detachment and cognitive dissonance.
3. **Dillon et al. (2018)**
   - **Summary**: Using survey data, Dillon and colleagues analyze surgeons’ attitudes toward death and dying, emphasizing how personal and professional beliefs influence delayed referrals to palliative care. They discuss “good death” and its implications for surgical practice (Dillon et al., 2018)​(dillon2018).
   - **Theories Used**: Theories on attitudes toward death and palliative care integration, cultural and personal beliefs affecting professional behavior.
4. **Arnold-Forster (2020)**
   - **Summary**: Arnold-Forster investigates the emotional toll of death in British operating theaters, highlighting how surgeons manage emotions through narratives and professional identity. The study argues that surgical culture often demands emotional suppression (Arnold-Forster, 2020)​(05 arnorld).
   - **Theories Used**: Narrative identity theory, emotional suppression, and professional identity in medical practice.
5. **Suwanabol et al. (2020)**
   - **Summary**: Focusing on surgical residents, this study reveals the gaps in training related to palliative and end-of-life care, with many residents facing stress and inadequacy when dealing with dying patients. The need for integrated palliative care training is emphasized (Suwanabol et al., 2020)​(suwanabol2020).
   - **Theories Used**: Learning and competency development theories in medical education, cognitive dissonance in underprepared professionals.
6. **Guest et al. (2011)**
   - **Summary**: This article addresses burnout and psychiatric morbidity among cancer surgeons, noting that personal relationships, workplace stress, and coping with patient suffering are significant stressors. The study advocates for organizational interventions to support surgeons (Guest et al., 2011)​(09 guest2011).
   - **Theories Used**: Burnout and workplace stress theories, organizational intervention for mental health.
7. **Donegan, Pasternack, & Kelly (2017)**
   - **Summary**: Donegan and colleagues explore the lack of formal training for surgeons in coping with patient death, arguing that the medical culture often discourages open discussions on emotions. They advocate for greater support systems to help surgeons cope with loss (Donegan et al., 2017)​(06 donegan).
   - **Theories Used**: Emotional intelligence and organizational support theory, suppression of emotional responses in high-stakes environments.
8. **Taylor et al. (2008)**
   - **Summary**: This paper examines the communication challenges surgeons face when discussing unexpected intraoperative deaths with families, highlighting the importance of structured communication strategies to support both family and surgeon well-being (Taylor et al., 2008)​(15 taylor2008).
   - **Theories Used**: Crisis communication theory, CARE and SHARE models in traumatic communication.
9. **Podnos & Wagman (2006)**
   - **Summary**: This article addresses the role of palliative surgery in alleviating patient suffering at end-of-life stages, detailing the ethical and emotional complexities surgeons face in such contexts (Podnos & Wagman, 2006)​(podnos2006).
   - **Theories Used**: Ethical theories in palliative care, quality of life and dignity in death.
10. **Maestre (2013)**

- **Summary**: The simulation study explores how simulated patient deaths affect surgical trainees, raising questions about stress, learning retention, and emotional impact. It emphasizes the role of simulation in training for real-life scenarios involving death (Maestre, 2013)​(17 maestre2013).
- **Theories Used**: Simulation-based learning and experiential learning theory, impact of stress on learning.

This synthesis provides an overview of the diverse perspectives and theories used to explore the effects of patient death on surgeons, highlighting the integration of psychological, organizational, and educational frameworks.

1. **Hinshaw (2005)**
   - **Summary**: This article explores spirituality in surgical palliative care, addressing the role of spiritual distress and the surgeon’s potential to alleviate it. Hinshaw emphasizes the “good death” and the need for a holistic approach to end-of-life care that includes spiritual dimensions, particularly in a culturally diverse society (Hinshaw, 2005)​(hinshaw2005).
   - **Theories**: Spirituality and meaning-making, total pain theory (Saunders), holistic and patient-centered care.
2. **Death and Life in Iraq (2005)**
   - **Summary**: This narrative reflects on the emotional toll experienced by surgeons in conflict zones, focusing on coping mechanisms in extreme circumstances. The article illustrates how trauma surgeons form a “collective consciousness” in dealing with mortality and use dark humor and camaraderie as coping strategies (Death and Life in Iraq, 2005)​(02 Deathandlifeiniraq).
   - **Theories**: Trauma and coping mechanisms, collective consciousness, camaraderie as resilience.
3. **Wiemann, Ketteler, & Fahy (2023)**
   - **Summary**: This study surveys surgeons' responses to patient death, highlighting limited training on coping mechanisms, and high burnout rates. Wiemann et al. argue for the inclusion of patient-loss management in medical education, especially due to increased mortality from COVID-19 (Wiemann et al., 2023)​(apm-12-01-70).
   - **Theories**: Burnout and emotional exhaustion, need for structured emotional education in medical training.
4. **Schecter (2011)**
   - **Summary**: Reflecting on personal experiences with mortality, Schecter’s essay discusses how the constant presence of death shapes a surgeon’s professional and personal life. He suggests that facing death directly may strengthen resilience but also calls for introspection and emotional support for surgeons (Schecter, 2011)​(schecter2011).
   - **Theories**: Mindfulness and presence in the face of death, resilience through direct experience with mortality, introspection as coping.
5. **Tarpley & Tarpley (2011)**
   - **Summary**: This article explores the spiritual dimensions of surgical palliative care, emphasizing the need for cultural sensitivity and understanding in end-of-life scenarios. It discusses surgeons’ responsibilities to respect patients’ spiritual needs and to navigate moral dilemmas compassionately (Tarpley & Tarpley, 2011)​(tarpley2011).
   - **Theories**: Spiritual and cultural sensitivity, moral distress, holistic approach in palliative care.
6. **Joliat, Demartines, & Uldry (2019)**
   - **Summary**: This systematic review examines the impact of patient death on surgeons, finding that many experience psychological morbidity such as PTSD. The article underscores the gap in training on how to manage patient death and advocates for more robust support systems (Joliat et al., 2019)​(04 joliat).
   - **Theories**: Psychological impact and PTSD in medical professionals, lack of training in emotional resilience, need for support structures.

**Summary of Theories**

The articles use diverse theoretical approaches to explore the effects of death and dying on surgeons. These include:

- **Burnout and Emotional Exhaustion**: Highlighting how regular exposure to patient death contributes to emotional fatigue (Wiemann et al., 2023)​(apm-12-01-70).
- **Spiritual and Holistic Care**: Addressing the spiritual needs of both patients and surgeons, particularly in palliative settings (Hinshaw, 2005; Tarpley & Tarpley, 2011)​(hinshaw2005)​(tarpley2011).
- **Coping Mechanisms and Resilience**: Collective resilience and camaraderie, especially in traumatic contexts, as well as the role of humor and mindfulness in facing mortality (Death and Life in Iraq, 2005; Schecter, 2011)​(02 Deathandlifeiniraq)​(schecter2011).
- **Total Pain Theory**: Incorporating physical, psychological, social, and spiritual aspects of pain in palliative care (Hinshaw, 2005)​(hinshaw2005).
- **Post-Traumatic Stress and Psychological Morbidity**: Exploring PTSD and stress responses among surgeons regularly exposed to patient death, with calls for better support and mental health training (Joliat et al., 2019)​(04 joliat).

These frameworks emphasize the complexity and emotional impact of mortality on surgeons and propose a need for supportive, holistic approaches to enhance their resilience and patient care skills.

4o

**You said:**

combine the two lists

**ChatGPT said:**

ChatGPT

**Combined Summaries and Theoretical Frameworks for Each Article**

1. **Hinshaw (2005)**
   - **Summary**: Explores spirituality in surgical palliative care, focusing on how surgeons can help alleviate spiritual distress in dying patients. Discusses the "good death" and holistic approaches that incorporate spiritual needs within diverse cultural contexts (Hinshaw, 2005)​(hinshaw2005).
   - **Theories**: Spirituality and meaning-making, total pain theory (Saunders), holistic and patient-centered care.
2. **Death and Life in Iraq (2005)**
   - **Summary**: Narrates the experience of surgeons in a conflict zone, illustrating how trauma surgeons develop coping mechanisms, such as collective resilience and camaraderie, to handle extreme scenarios involving mortality (Death and Life in Iraq, 2005)​(02 Deathandlifeiniraq).
   - **Theories**: Trauma and coping mechanisms, collective consciousness, camaraderie as resilience.
3. **Wiemann, Ketteler, & Fahy (2023)**
   - **Summary**: This survey-based study investigates how surgeons respond to patient death, revealing that many lack training in coping strategies and experience burnout. The study emphasizes the need to integrate loss management and emotional resilience training into medical education (Wiemann et al., 2023)​(apm-12-01-70).
   - **Theories**: Burnout and emotional exhaustion, need for structured education on handling patient death.
4. **Schecter (2011)**
   - **Summary**: Schecter’s reflection on mortality in surgery highlights the role of direct experience with death in building resilience. He argues for introspection and mindful engagement with mortality to strengthen surgeons’ emotional endurance and adaptability (Schecter, 2011)​(schecter2011).
   - **Theories**: Mindfulness and presence in facing death, resilience through direct experience, introspection as a coping mechanism.
5. **Tarpley & Tarpley (2011)**
   - **Summary**: Focuses on the spiritual dimensions of surgical palliative care, stressing the importance of respecting diverse cultural and spiritual needs in end-of-life care. Discusses the role of surgeons in navigating ethical dilemmas and addressing moral distress compassionately (Tarpley & Tarpley, 2011)​(tarpley2011).
   - **Theories**: Spiritual and cultural sensitivity, moral distress, holistic approach in palliative care.
6. **Joliat, Demartines, & Uldry (2019)**
   - **Summary**: This systematic review reveals the psychological toll of patient death on surgeons, including symptoms of PTSD and burnout. Joliat et al. advocate for improved training and support structures to help surgeons manage the emotional impact of patient death (Joliat et al., 2019)​(04 joliat).
   - **Theories**: Psychological impact and PTSD in medical professionals, emotional resilience training, need for support structures.

**Summary of Key Theories Across Articles**

- **Burnout and Emotional Exhaustion**: Chronic exposure to death contributes to emotional fatigue among surgeons, underscoring the need for coping strategies (Wiemann et al., 2023)​(apm-12-01-70).
- **Spiritual and Holistic Care**: Emphasizes the importance of addressing spiritual needs and cultural sensitivity in palliative care for both patients and surgeons (Hinshaw, 2005; Tarpley & Tarpley, 2011)​(hinshaw2005)​(tarpley2011).
- **Coping Mechanisms and Resilience**: Collective resilience, camaraderie, and humor serve as coping methods for surgeons in traumatic settings, and mindfulness enhances resilience (Death and Life in Iraq, 2005; Schecter, 2011)​(02 Deathandlifeiniraq)​(schecter2011).
- **Total Pain Theory**: Incorporates physical, psychological, social, and spiritual pain, advocating for a holistic approach in palliative care (Hinshaw, 2005)​(hinshaw2005).
- **Post-Traumatic Stress and Psychological Morbidity**: High rates of PTSD and psychological stress in surgeons call for better mental health training and support structures (Joliat et al., 2019)​(04 joliat).

Together, these frameworks illustrate the profound impact of mortality on surgeons and underscore the need for supportive, multidimensional approaches to foster resilience and effective patient care.
