# Supplementary material for: The impact of death and caring for the dying and their families on surgeons - an AI assisted systematic scoping review
Source: BMC Surg. 2025 Feb 5;25:56. doi: 10.1186/s12893-025-02792-1 (PMC11796083; doi:10.1186/s12893-025-02792-1)
Supplement: Supplementary file 5 — Supplementary Material 5 [file 12893_2025_2792_MOESM5_ESM.docx]

| **Article Title** | **Impact of Death and Dying** | **Workload and Working Hours** | **Frequency of Exposure to Death and Dying** | **Duration of Care or Relationship with Patients** |
| --- | --- | --- | --- | --- |
| **A Piece of My Mind: Death and Life in Iraq** | Emotional toll in trauma surgery | Non-stop, intense combat zone work | High frequency in war zone trauma | Brief, dictated by trauma urgency |
| **A Small Cemetery: Death and Dying in the Contemporary British Operating Theatre** | Persistent psychological burden in neurosurgery | Focus on efficiency limits emotional processing | Regular exposure in neurosurgery | Prolonged for complex cases |
| **Are Surgeons Capable of Introspection?** | Surgeons' resistance to introspection | High workload limits introspection | Frequent exposure in palliative surgery | Episodic but emotionally lasting |
| **A Surgeon's Reflections on the Care of the Dying** | Shift in surgical philosophy towards palliative care | Workload pressures inhibit introspection | Routine in cancer and advanced illness | Prolonged in palliative cases |
| **Cancer Surgeons' Distress and Well-being** | High distress linked to cancer patient deaths | High workload contributes to burnout | Frequent in oncological surgery | Long-term in cancer care |
| **How Prepared Are Surgical Foundation Year One Doctors to Deal with Patients in the Last Days of Life** | Gaps in palliative training for junior doctors | Heavy workloads limit palliative care focus | Common, varies by hospital type | Short-term, mostly acute care |
| **Is a Foundation Interim Year One Post Better for Improving Confidence in the Care of the Dying** | Confidence disparities in palliative care training | Variable workloads based on hospital type | Frequent in tertiary centers | Varies, more extended in tertiary centers |
| **Deciphering the Surgeons' Stories** | Emotional narratives highlight ethical dilemmas | Emotional toll compounded by workload | Frequent across surgical specialties | Short-term but emotionally intense |
| **Euthanasia as an Example of Conflict Between Ethical Precepts and Entitlement Rights** | Ethical complexities around euthanasia | Emotional workload from ethical dilemmas | Regular in terminal illness cases | Extended in terminal cases |
| **Surgeon Perspectives Regarding Death and Dying** | Delays in palliative referrals and emotional toll | Time constraints limit end-of-life care | Frequent, especially near death | Limited, mostly perioperative care |
| **Lessons in Death and Dying on an East African Surgical Ward** | Cultural differences in palliative care | High patient burden in low-resource settings | Frequent due to resource limitations | Short, pragmatic care |
| **Meditation on Mortality: Lessons from a Life in Surgery** | Emotional reflections on mortality | Long-term emotional involvement | Regular exposure to terminal patients | Long-term patient relationships |
| **Reflections About Death in the Surgical Simulation Environment** | Emotional impact of simulated deaths | Simulation hours mirror real-world surgery | Occasional but impactful in simulations | N/A – simulation-based |
| **Residency Diary: The Gift of Learning** | Emotional growth through cadaver work | Long hours of residency work | Routine cadaver exposure | Months-long cadaver work |
| **SAGES Primer for Taking Care of Yourself During and After the COVID-19 Crisis** | Burnout and trauma from COVID-19 deaths | Overwhelming workloads during pandemic | High mortality during pandemic | Brief, acute care focus |
| **Spiritual Dimensions of Surgical Palliative Care** | Spiritual engagement in palliative care | Emotional and spiritual burdens add to workload | Frequent in palliative surgery | Extended in palliative contexts |
| **Spiritual Issues in Surgical Palliative Care** | Emotional burden due to surgical “intimacy” between surgeon-patient dyad | Not Applicable | Not Applicable | Extended in palliative contexts |
| **Surgeon and Medical Student Response to Patient Death** | Burnout and emotional distress in medical students | High workload contributes to burnout | Frequent, varies by specialty | Short-term, perioperative care |
| **Surgery Residents' Experiences With Seriously-Ill and Dying Patients** | Lack of structured training in palliative care | Residents report heavy clinical workloads | High exposure among residents | Limited to inpatient settings |
| **Systematic Review of the Impact of Patient Death on Surgeons** | Significant stress and psychological toll | Long hours exacerbate stress | Frequent in trauma and cancer surgery | Procedural, limited duration |
| **The Death of Daniel Prude – Reflections of a Black Neurosurgeon** | Intersection of systemic racism and medical care | Emotional labor adds to clinical responsibilities | Occasional but emotionally significant | Limited but deeply personal |
| **The Surgeon and Palliative Care** | Growing role of palliative surgery | Palliative surgery adds to workload | Frequent in cancer care | Prolonged for palliative patients |
| **The Surgeon-Patient Relationship in Advanced Illness** | Importance of empathy in advanced illness | Emotional labor in advanced illness care | Frequent in advanced illness | Prolonged engagement |
| **Unexpected Intraoperative Patient Death** | Surgeon trauma after intraoperative deaths | Surgeons feel unskilled in death communication | Rare but significant intraoperative deaths | Brief, with family follow-up |
| **What We Still Don’t Know About Surgeon Response to Patient Death** | Burnout due to patient deaths | Heavy workload reduces emotional processing | Frequent in trauma and critical care | Episodic, limited continuity |
| **Your Best Life – Dealing with Loss** | Orthopaedic surgeons underprepared for death | Time pressures limit emotional response | Rare but impactful in orthopaedics | Short but emotionally significant |
